# Current Status of the Diagnosis and Treatment of Mismatch Repair Deficient Colorectal Cancer

**DOI:** 10.3390/biomedicines14051032

**Published:** 2026-05-01

**Authors:** Donald J. Bastin, Vladimir Djedovic, Angela Hyde, Rachel A. Goodwin, Timothy R. Asmis, Michael M. Vickers

**Affiliations:** 1 Division of Medical Oncology, Department of Medicine, The Ottawa Hospital, The University of Ottawa, Ottawa, ON K1N 6N5, Canada; dbastin@toh.ca (D.J.B.); vdjedovic@toh.ca (V.D.); rgoodwin@toh.ca (R.A.G.); tasmis@toh.ca (T.R.A.); 2 Cancer Care Program and Laboratory Medicine Program, NL Health Services, St. John’s, NL A1B 3X5, Canada; angela.hyde@nlhealthservices.ca

**Keywords:** immunotherapy, mismatch repair deficiency, colorectal cancer, microsatellite instability, pathogenesis, molecular testing

## Abstract

Colorectal cancer remains a leading cause of morbidity and mortality worldwide with diverse pathways of carcinogenesis. Deficiencies in the DNA mismatch repair and resultant microsatellite instability are thought to make up roughly 15% of localized and 5% of metastatic cancers of the colon and rectum. Cancers arising through this pathway are characterized by poor response to traditional chemotherapies, but have demonstrated unprecedented responses to immunotherapy over the last decade. Thus, the management of mismatch repair-deficient/microsatellite-unstable colorectal cancer is a rapidly evolving field. In this review we provide a clinician-oriented update on the diagnosis and management of mismatch repair-deficient/microsatellite-unstable colorectal cancer. We explore the tools used for diagnosis as well as the causes and implications of the failure of these tools, along with practical recommendations to mitigate and circumvent such errors. Furthermore, we examine the changing treatment paradigm in the advanced setting with the implementation of mono and dual immunotherapy approaches and explore who is most likely to benefit from such strategies, and how to address treatment failures. Finally, we explore how immunotherapy may allow for non-surgical approaches in the localized setting and discuss the evolving evidence for neoadjuvant and adjuvant approaches when surgery is used.

## 1. Introduction

Colorectal cancer (CRC) is now the third most commonly diagnosed cancer worldwide and ranks second in terms of cancer mortality [1]. While new cases in older adults are declining, both the incidence and mortality in younger patients are rising [2]. Furthermore, CRC is becoming increasingly common in countries with low human development index therefore, the overall burden of the disease is increasing [1,2].

CRC is a biologically heterogeneous disease with various distinct but sometimes overlapping pathways of carcinogenesis [3,4]. This diversity has implications for both the natural history of a patient’s disease and the response to treatment. Treatment options are increasingly tailored to individual disease biology, and there are now a multitude of biomarkers required to establish the optimal individualized treatment of CRC [5].

One such pathway involves deficiencies in DNA mismatch repair (MMR) proteins. MMR deficiency (dMMR) is thought to underline approximately 15% of localized cancers of the colon and rectum, as well as 5% of metastatic colorectal cancers [6,7,8]. dMMR portends a worse response to chemotherapy when compared to tumors that are MMR proficient (pMMR) both in the localized and metastatic settings [9,10,11]. However, the introduction of immunotherapy has drastically changed treatment guidelines and outcomes for dMMR CRC, and of this evidence finding is rapidly evolving [12,13,14]. In this review, we will summarize the basic science as well as clinical considerations when testing for dMMR in CRC. Furthermore, we will explore the most recent developments in the use of immunotherapy for dMMR CRC in both the localized and metastatic settings.

## 2. Mismatch Repair Deficient/Microsatellite Unstable Colorectal Cancer

### 2.1. Biology of dMMR and Microsatellites

Microsatellites are short, repetitive, non-coding sequences of DNA that are found throughout the human genome. DNA replication can be faulty in these areas when machinery “slips” on repeat nucleotides, causing addition or loss of nucleotide repeats and a “mismatch” between the length of the parent and daughter strands [15,16]. The term “microsatellite instability” (MSI) was coined in 1993 to describe the observation of alterations in the length of microsatellite sequences of certain CRC samples as a result of such mismatches [17,18,19]. Alterations in the lengths of microsatellites can cause frameshift mutations that activate proto-oncogenes or inactivate tumor suppressors, contributing to carcinogenesis [19,20,21].

**Figure 1:**
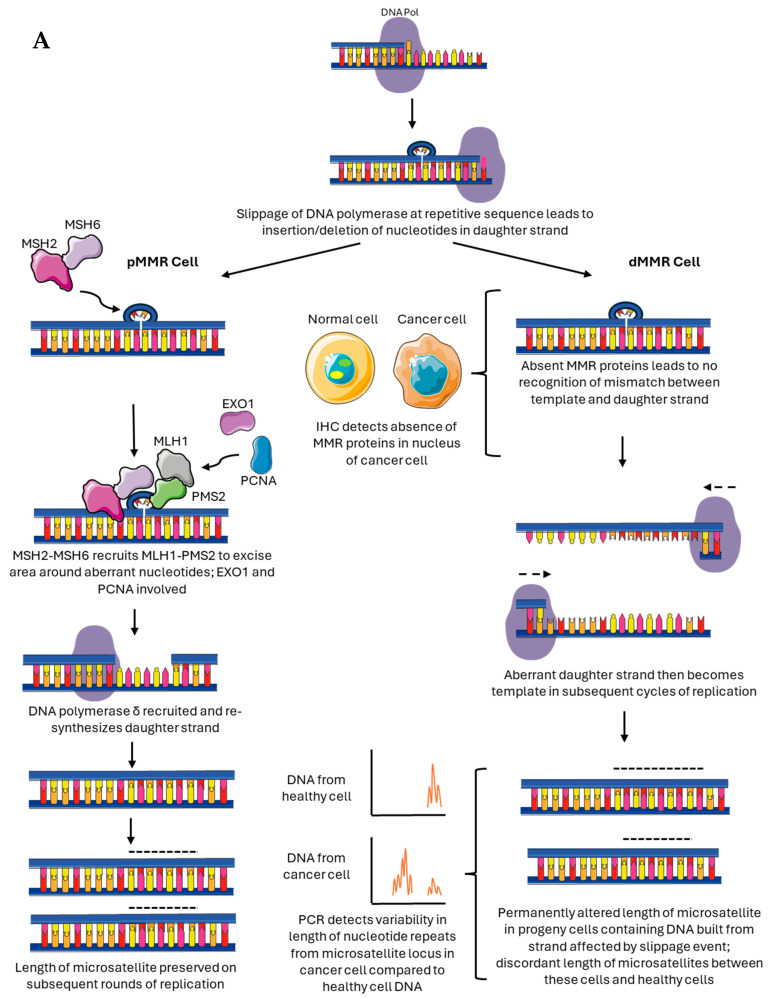

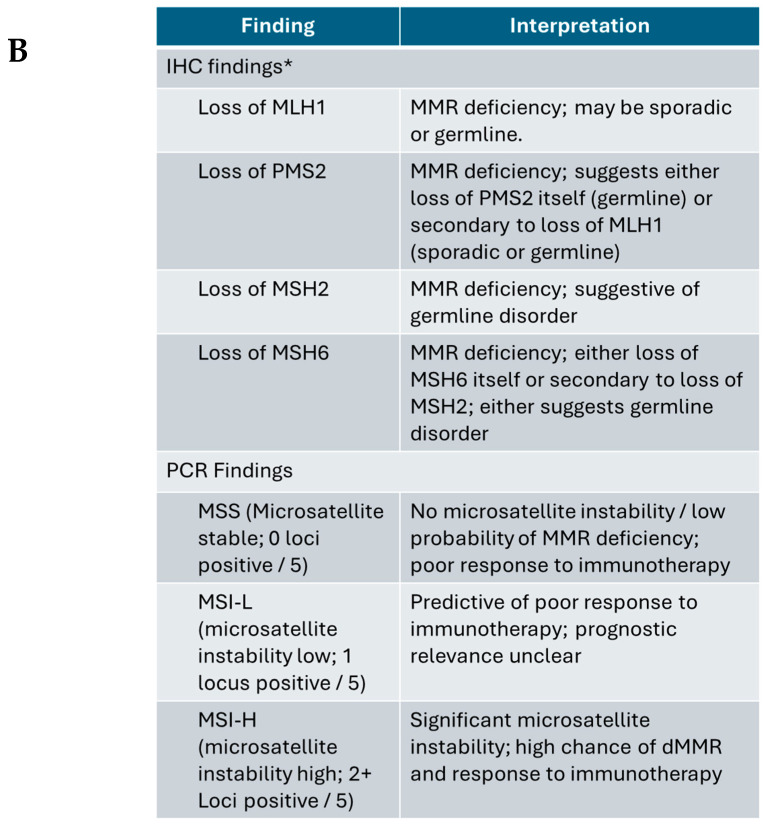
(**A**) Mechanisms of mismatch repair in healthy cells and of microsatellite instability in mismatch repair-deficient cells along with detectable consequences by immunohistochemistry and polymerase chain reaction. DNA polymerase is error-prone in microsatellite areas due to slippage on repeat nucleotides, leading to the addition of nucleotides or truncation of the daughter strand. Under normal circumstances, errors are detected by the MSH2-MSH6 complex, which then recruits MLH1-PMS2, which interacts with other proteins to remove the erroneous nucleotides. DNA polymerase δ re-synthesizes the daughter strand. In cases of loss of the MMR proteins, the erroneous daughter strand is preserved and serves as a template in subsequent rounds of replication. This results in permanent alterations in the length of microsatellites being incorporated in the progeny of cells affected by the initial slippage event. Deficiencies in mismatch repair proteins can be detected by immunohistochemistry, while alterations in the length of microsatellites (i.e., microsatellite instability) can be detected by PCR. Images adapted from Servier Medical Art (https://smart.servier.com accessed 27 January 2026), licensed under CC BY 4.0 (https://creativecommons.org/licenses/by/4.0/). (**B**) Interpretation of testing for mismatch repair deficiency and microsatellite instability. Staining patterns on IHC and the number of microsatellites affected can provide clues to disease biology. * All IHC findings listed imply a high chance of microsatellite instability and a good response to immunotherapy.

Under normal circumstances, the MMR machinery within a cell, comprising proteins MLH1, MSH2, MSH6, PMS2, and others, is adept at recognizing and repairing DNA damage. In brief, MSH2-MSH6 (or MSH2-MSH3) heterodimers search the genome and recognize the 3-dimensional shape changes caused by aberrant pairings in the DNA [22,23]. When they encounter such a mismatch, the MLH1-PMS2 complex is recruited in a process that hydrolyzes ATP [24,25,26]. MLH1 and MSH2 form the dominant components of their respective dimers, without which the other proteins are degraded [15]. PMS2 interacts with several other proteins, including EXO1 and PCNA to remove the erroneous DNA sequence [26,27,28]. A new daughter strand is then synthesized by DNA polymerase δ using the original parent strand as a template [23,29,30]. The MMR pathway is also involved in inhibiting cell cycling in situations where repair cannot be achieved [31,32]. The MMR process has been reviewed in great detail elsewhere, and it, along with the implications of the test results, is summarized in Figure 1 [23,26,32,33].

In dMMR CRC, aberrations in the MMR pathway occur as germline alterations in roughly 20% of cases and arise spontaneously about 80% of the time [34]. Lynch syndrome is the most common cause of heritable dMMR CRC and underlies roughly 3% of CRC diagnoses. It is most frequently attributed to germline heterozygous loss of MLH1 or MSH2, although loss of other MMR proteins as well as epithelial cell adhesion molecule (EpCAM) has also been implicated [35]. These mutations predispose patients to full loss of MMR capacity through a “second hit.” Phenotypically, this causes an earlier median age of onset of CRC (45 years), risk of multiple colon cancers, a predominance of right-sided and higher-grade CRCs, as well as a heightened risk of other malignancies including endometrial and urinary tract cancers [35,36]. Associations have been observed between the specific gene mutations and cancer syndromes, with MLH1 loss having the highest incidence of CRC and MSH6 being more associated with endometrial cancer for example [36,37]. Less commonly, homozygous germline loss of MMR proteins can occur as a constitutional mismatch repair deficiency syndrome (CMMR-D) which is associated with other cancers, including CNS tumors and hematologic malignancies, in addition to Lynch-associated tumors [26,38].

Conversely, the vast majority of somatic cases of dMMR CRC arise through epigenetic silencing of MLH1 [26,39,40,41]. CpG island methylation (CIMP) is a recognized pathway to CRC, and tumors exhibiting this gene signature have been shown to cluster with the MSI gene signature [4]. Mutations in BRAF, most notably V600E, have also been linked to CIMP and are enriched in sporadic dMMR CRC [42,43]. BRAF mutations are able to trigger and maintain promoter methylation, suggesting that these could be early and critical events in the pathogenesis of some dMMR colorectal cancers [44,45]. However, MLH1 promotor methylation and sporadic dMMR CRC have been observed in the absence of BRAF mutation, suggesting that it is not a necessary event along the pathway to carcinogenesis [46]. Other well-described events, including mutations in KRAS and NTRK fusions have been detected in a high proportion of dMMR CRC samples that exhibit MLH1 methylation but are wild type for BRAF [46,47]. The mutational profile of sporadic dMMR CRC can have prognostic and predictive implications. For example, BRAF-mutated dMMR CRC has been shown to have a worse prognosis than BRAF-WT dMMR disease, but a superior prognosis to BRAF-mutated pMMR disease [48,49]. Both BRAF and NTRK are targetable mutations in later lines of therapy [5].

While dMMR/MSI-H can drive carcinogenesis, it also underpins the generation of neoantigens through frameshift mutations, which can, in turn, provide a substrate for immune recognition of the tumor [50]. The frequency of mutations can be described as tumor mutational burden (TMB), with dMMR CRC being generally associated with higher TMB than pMMR disease and higher TMB being associated with better outcomes [51]. Along these lines, neoantigen load in dMMR/MSI-H CRC has been shown to correlate with frequency of tumor infiltrating lymphocytes (TILs) [52]. The theory of immunoediting postulates that while these TILs may initially mediate tumor rejection and immunity, clinically apparent tumors arise through adaptation of the cancer cells and resistance to immune killing through mechanisms such as upregulation of immune checkpoints, downregulation of MHC molecules, and modulation of the microenvironment [53]. This provides a biological rationale for the responsiveness of dMMR/MSI-H CRC to immunotherapies, which seek to tip the balance back in the favor of the immune system.

### 2.2. Clinical Testing for MMR/MSI Status

Owing to the prognostic and therapeutic implications, it is now widely recommended that all patients with a diagnosis of CRC undergo testing for dMMR and/or MSI at diagnosis [54,55,56]. A summary of testing strategies can be found in Table 1. The most common mechanism for testing for dMMR is through immunohistochemistry (IHC) and this has been endorsed by several national bodies [15,54,55,56]. IHC is often the quickest and most readily available testing modality and provides a high degree of accuracy provided that testing is done for all four proteins, MLH1, MSH2, MSH6, and PMS2 to avoid false negatives [15,55,57]. The sensitivity of IHC is quoted as being similar to MSI testing at 92–94% [58,59]. It should be noted that following treatment, in particular radiotherapy, expression of MMR proteins can be lost by IHC. Where possible, testing should be done on pre-treatment specimens [60]. IHC can be performed either on biopsied or surgically resected specimens. The former offers more timely results, superior formalin fixation, and the possibility of using neoadjuvant treatment in the localized setting; while the latter provides a larger sample to choose from and can better assess background control and intratumoral heterogeneity [54].

Polymerase chain reaction (PCR) to detect MSI is recognized as an acceptable alternative or complementary test to assessing dMMR by IHC [54,55,56]. The originally established PCR-based method for assessing MSI involves testing five mononucleotide loci commonly affected in MSI CRC known as the Bethesda panel [15,64]. Based on the revised Bethesda guidelines, a tumor is called MSI-high (MSI-H) if two or more of the loci are positive, MSI-low (MSI-L) if one is positive, and microsatellite stable (MSS) if none are positive [64]. In general, the MSI-L subset is not felt to respond well to immunotherapy and some guidelines advocate that MSI-L should be labeled as MSS [55,65]. Although seemingly not predictive of the response to immunotherapy, some evidence suggests that MSI-L may nevertheless have prognostic implications and be associated with a distinct clinicopathological group from MSS, but this finding is controversial [66]. The Bethesda panel has been shown to have a sensitivity of 91.7% and specificity of 98.4%, with newer commercial panels having even higher sensitivities and specificities depending on the study [62,63,67]. The European Society of Medical Oncology (ESMO) now recommends the five poly-A mononucleotide panel as it has a slightly higher sensitivity of 95.6% [55,62].

**Figure 2:**
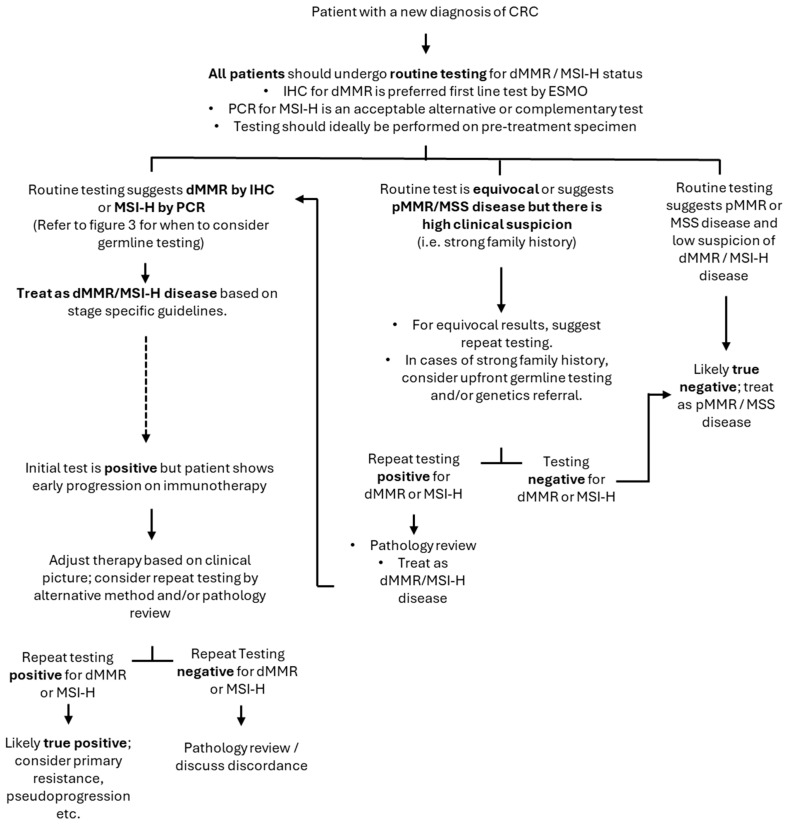
Clinical workflow for approach to testing for mismatch repair-deficiency/microsatellite-instability in colorectal cancer (adapted from the guidelines by Bartley et al. [54], Luchini et al. [55], and Vikas et al. [56]). A new diagnosis of colorectal cancer should prompt assessment for dMMR/MSI-H status either with IHC or PCR. ESMO suggests IHC as a first-line test, although either one is acceptable. There is currently a limited role for the NGS outside of research or specialized settings. Initial testing suggestive of dMMR/MSI-H disease should prompt treatment for the dMMR/MSI-H disease as well as the assessment of affected protein and the assessment of MLH1 promotor status and/or BRAF status as appropriate. Refer to Figure 3 for the approach to germline testing. In the event of dMMR/MSI-H disease on initial testing but an unexpected response to immunotherapy, adjust therapy based on clinical response and consider repeat testing by an alternative method to rule out a false positive result. If the initial test is equivocal, follow-up testing should be strongly considered. If initial testing is suggestive of pMMR/MSS disease, but there is a strong family history, germline testing or genetics referral should still be considered. Other clinical factors may also play into the decision to pursue additional testing, although false negatives on initial testing are uncommon. In the event of an initial negative test and no compelling clinical reasons to suggest otherwise, this is likely a true negative result, and the clinician should consider treating it as pMMR/MSS disease.

Owing to accessibility, ESMO recommends that IHC be used as a first-line test with the PCR-based testing being used for confirmation or in the event of equivocal or uninterpretable IHC results [55]. The American Society of Clinical Oncology (ASCO) and the College of American Pathologists (CAP) recommend that either IHC or PCR be used as a first-line test, but similarly recommend the use of an alternative test in the case of inconclusive initial results [54,56]. Presently, the incidence of discordance between tests is felt to be low, with one study showing a discordance rate of only 0.4%, suggesting that such a strategy is probably reasonable as a general practice [15,68]. Nevertheless, there are documented cases of MMR/MSI testing producing inaccurate results, and these can have important consequences. Figure 2 represents our suggested approach to clinical workflow MMR/MSI testing in CRC.

### 2.3. Clinical Testing Implications of Sporadic vs. Inherited dMMR?

Identification of patients harboring germline alterations in MMR machinery is critical, as it has implications both for screening of the patients themselves and in terms of risk management of potentially affected family members. These guidelines are summarized elsewhere [69]. Patients should be selected for germline testing in a way that balances the need to identify those with a high probability of having a germline dMMR syndrome with responsible resource utilization and the avoidance of over testing.

**Figure 3:**
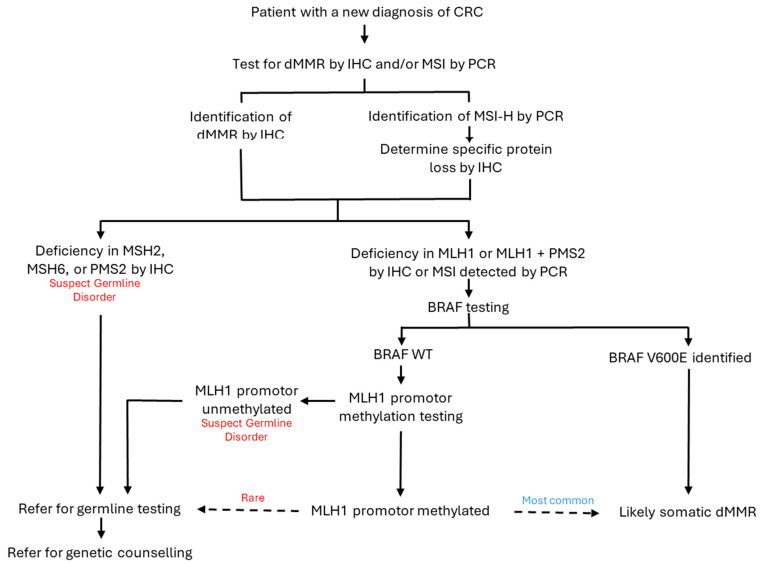
Proposed pathway for determination of somatic vs. germline loss of MMR proteins (adapted from NHS 2023 [74] and Bartley et al. 2022 [54]). Any patient with a new diagnosis of CRC should be tested for dMMR by IHC or MSI by PCR. If dMMR or MSI is detected, the patient should go to screening for a germline condition. Patients in whom MSI was detected by PCR should have an assessment of MMR proteins by IHC. Deficiencies in MSH2, MSH6, or PMS2 by IHC should automatically prompt suspicion of a germline disorder. In patients with an absence of MLH1 or MLH1 + PMS2, BRAF testing should be carried out. BRAF V600E makes a germline disorder highly unlikely, while WT BRAF indicates the possibility of a germline disorder. MLH1 promoter testing can be carried out to further clarify where an unmethylated promotor suggests a germline disorder. The presence of MLH1 methylation is highly suggestive of somatic dMMR, but cannot entirely rule out a germline disorder and should be taken in light of the clinical context and any additional information. Rare cases of MLH1 hypermethylated germline conditions have been described, and testing for constitutional MLH1 promoter hypermethylation can be considered to further informof the risk of a germline disorder in select cases.

As implied by the previous section, the pattern of loss of MMR proteins on IHC can provide clues to whether dMMR has occurred due to somatic or germline mechanisms. Loss of proteins other than MLH1 is typically more suggestive of a germline syndrome. MLH1, however, can be lost by both germline and somatic mechanisms, and the fact that the loss of a dominant binding partner (MLH1 or MSH2) leads to the absence of its’ heterodimer (PMS2 or MSH6) further complicates interpretation, highlighting the importance of testing all four proteins [15,54]. Identification of MLH1 methylation by methylation testing is suggestive of sporadic, rather than germline MLH1 loss; however, rare instances of MLH1 promoter methylation in germline dMMR CRC have been described, particularly in younger patients [70,71]. Constitutive MLH1 promotor hypermutation may be the cause of approximately 3% of the cases of Lynch syndrome [72]. Concomitant mutation of BRAF in the context of dMMR CRC is a much stronger predictor of sporadic as opposed to germline dMMR [73]. Thus, current guidelines suggest an iterative approach to identifying individuals for germline testing. Patients with a loss of MSH2, MSH6, or PMS2 should be referred for germline testing, as should those who have a loss of MLH1 in the absence of promoter methylation. A BRAF V600E mutation usually signals against germline dMMR, but an MLH1 promoter methylation is usually, but not always, associated with sporadic dMMR. Thus, in patients where there is a strong clinical suspicion for germline loss of MMR proteins, this should not preclude referral for germline testing [54,74]. A proposed algorithm for when to refer for germline testing can be found in Figure 3.

### 2.4. Accuracy of dMMR/MSI Testing

The concordance between MMR and MSI testing is generally high based on studies comparing MMR to MSI testing within individual institutions [15,68,75]. However, in both the CheckMate-142 and CheckMate-8HW trials, patients that were identified as being dMMR or MSI-H based on local testing were found to have MSI-L or MSS disease on centralized testing in 15–20% of cases [76,77,78]. Interestingly, in CheckMate-142, 21% patients who were pMMR on central testing, but dMMR on local testing did in fact respond to treatment, suggesting that the central testing may produce false negatives in some cases [76]. One caveat was that the local and centralized testing were not necessarily performed on the same biopsy specimen. However, it is unlikely that the tumor as a whole would have changed MMR expression or MSI status over that time in all of these cases [76]. In a post hoc analysis of 38 patients enrolled in immunotherapy trials for metastatic dMMR/MSI-H CRC at a single center, authors found that out of 5 patients who experienced hyperprogression, 3 (60%) were pMMR/MSS on repeat testing [79]. Taken together, these results suggest that MMR/MSI testing can produce inaccurate results in a high proportion of patients, on the order of 20%, even in the context of large-scale clinical trials, and that these errors can have important clinical consequences.

The biological causes of inaccurate test results for dMMR/MSI-H CRC have been more extensively reviewed elsewhere but will be summarized here as they pertain to clinical decision-making [63,80]. Several studies have documented intratumoral heterogeneity both in loss of MMR proteins and in alterations at specific microsatellite loci on repeat testing of the same tumor sample [81,82,83]. While dMMR heterogeneous tumors may have a poorer response to immunotherapy, preclinical studies and case reports have documented situations where these tumors can respond to immunotherapy, implying that MMR heterogeneity can yield both false positives and false negatives when being used to predict response to immunotherapy [84,85,86]. Dysfunctional MMR proteins that retain IHC staining have been implicated as a cause of false positives, while functional overlap between MSH6 and MSH3 as well as weak MMR protein expression that retains mismatch repair activity can cause false negatives by IHC [63,80,87]. Specific variant alleles that have the appearance of unstable microsatellites have been shown to produce false positives by MSI-PCR, while the contamination of the tumor samples with non-tumor cells can yield false negatives [79,88]. Finally, technical issues, including sample preparation and interpretation of ambiguous staining patterns on IHC can also be implicated in the failure of tests to accurately identify dMMR/MSI-H tumors that may benefit from immunotherapy [63,89].

Although ESMO guidelines recommend a tiered approach with MMR testing by IHC followed by MSI testing with PCR in the setting of indeterminate IHC results, they make a specific comment recommending both tests when assessing for treatment with immunotherapy due to the aforementioned large number of false positives in some trials [55]. Balancing the critical importance of accurate diagnosis with the implications of requesting additional tests, particularly in resource-constrained settings, can be challenging. We would argue that given the high number of false positives observed between local and centralized testing in the CheckMate trials, it may be reasonable to adopt a policy whereby patients who demonstrate early progression are re-tested to ensure a reasonable expectation that their disease would respond to immunotherapy [76,77,78]. It is equally important to ensure that patients who do in fact have dMMR/MSI-H do not lose access to potentially effective therapy due to false negatives. Thus, history and clinical suspicion are important considerations when interpreting MMR/MSI results and may prompt further discussions between treating physicians and pathologists [54]. Emerging work suggests that the presence of high-risk characteristics such as right-sided tumors and the PIK3CA mutation should prompt additional levels of testing even when initial tests suggest pMMR/MSS disease [90]. Inaccuracies in the diagnostic tools for MMR/MSI represent an under-appreciated issue in clinical practice, and further work in optimizing testing is important. This also highlights the need for good quality assurance within existing practices.

Next-generation sequencing (NGS), which employs deep sequencing (i.e., multiple reads of a given area of the genome) to detect alterations in lengths of microsatellites while concurrently identifying key mutations and quantifying tumor mutational burden, has been proposed as a superior mechanism for detection of dMMR [55,91]. While ESMO guidelines suggest that a validated NGS test may be an acceptable alternative to IHC/PCR testing, the CAP/ASCO recommendations are that it should only be used in highly selected cases [54,55,56]. The primary reasons for this include the significant cost, technical expertise, additional tumor tissue/DNA, and time to return the results that are associated with NGS [54]. However, ESMO acknowledges that with advancements in NGS technology, this may become the preferred testing modality in the future [55]. Indeed, NGS platforms with superior ability to detect true dMMR CRC, compared to IHC methods have been described recently. Therefore, it is possible that in further iterations of NGS testing some of the logistical and cost issues will make this a viable technique for more widespread use [92].

## 3. Management of Metastatic dMMR/MSI-H CRC

### 3.1. State of the Art in Immunotherapy for Metastatic dMMR/MSI-H CRC

Key trials outlining milestones in the treatment of metastatic dMMR/MSI-H CRC are summarized in Table 2.

dMMR/MSI-H is historically associated with a poorer prognosis and response to chemotherapy in the metastatic setting [9]. Studies in the early 2010s exploring the use of programmed death 1 (PD-1) inhibition in unselected metastatic CRC demonstrated an acceptable safety profile, but an underwhelming response rate (RR) with a single observable (albeit complete) response [93,94]. In 2015, Le et al. hypothesized that this patient’s tumor had in fact been dMMR and reported a subsequent 41 patient trial showing a response rate of 40% for patients with metastatic dMMR CRC treated with pembrolizumab compared to 0% in those with pMMR disease [95]. This led to the seminal CheckMate-142 and Keynote-164 trials, which showed objective responses of 39% (31.1% in the initial report) and 33% to single agents nivolumab and pembrolizumab, respectively, in patients with previously treated dMMR CRC [76,96,97].

Keynote-177 was a large phase III trial that randomized previously untreated dMMR/MSI-H mCRC patients to either pembrolizumab or investigator’s choice chemotherapy (mFOLFOX6 or FOLFIRI +/− bevacizumab or cetuximab). Pembrolizumab showed improvements in progression-free survival (PFS) and health-related quality of life and an unprecedented 77.5 month median overall survival in the experimental group compared to 36.7 months with chemotherapy. In addition, the response rate to pembrolizumab was 45.8% (versus 33.1%), including a complete response (CR) rate of 17.6%, and there was a significantly lower rate of grade 3–5 toxicities in favor of pembrolizumab (22% vs. 67%). While the overall survival did not meet statistical significance at the final planned analysis, this was felt to be due to a high crossover rate of 62%. Despite these practice-changing results, a concerning finding was that 30.1% of patients in the pembrolizumab group had progressive disease (PD) as their initial response [98,99,100]. This is consistent with PD in 40–46% of patients in Keynote-164 and 21% of patients in the nivolumab monotherapy cohort from CheckMate-142 [76,96,101]. To improve response rates, studies have investigated the addition of cytotoxic T-lymphocyte-associated protein 4 (CTLA-4) inhibition to PD-1/PD-L1 axis inhibition (“dual immunotherapy”). CheckMate-142 was a multicohort study that included a cohort of 45 patients who received nivolumab and ipilimumab in the first-line setting. This reported an overall response rate of 71% with only 13% of patients showing PD at initial follow-up [97,102]. At a median follow-up of 52.4 months, the median survival in the dual immunotherapy cohort of CheckMate-142 has not been reached [97]. Expanding on this theme, the CheckMate-8HW trial randomized untreated or previously treated metastatic dMMR/MSI-H CRC patients 2:2:1 to either nivolumab plus ipilimumab, nivolumab alone, or the investigator’s choice of chemotherapy with or without targeted therapies. Previously, nivolumab + ipilimumab showed significant improvements in PFS compared with chemotherapy, and in a recent interim analysis, the dual immunotherapy arm showed an impressive response rate of 71% (30% CR) compared to 58% (with 28% CR) for nivolumab alone [77,78]. Although the survival data for this trial is immature, median PFS had not been reached after a 47-month follow-up in the dual immunotherapy group compared to 39.3 months in the nivolumab alone group. Importantly, the rate of primary progressive disease in the dual immunotherapy arm was 10% [77].

Current guidelines recommend pembrolizumab in the first line for patients with metastatic dMMR/MSI-H CRC; however, the nivolumab + ipilimumab combination has recently been approved by the Food and Drug Administration (FDA) and the National Institute for Health and Care Excellence (NICE) for this indication [12,14,103,104]. It should also be noted that immunotherapy has been linked to improved health-related quality of life when compared to chemotherapy and has been shown to be cost effective [77,105,106].

**Table 2:** Key Trials of Immunotherapy in Patients with Metastatic dMMR/MSI-H Colorectal Cancer. References [76,77,78,96,97,98,99,100,101,102,107,108,109,110].

Trial (Enrollment Start Date)	Study Design	Therapy Line	Treatment Groups								
			Treatment	F/U (mo)	ORR	OS	PFS	DOR	PD	AE 3–4	Key Findings
CheckMate-142 (14 March 2014) [76,97,102,108,109,110]	Phase II, multicenter, open-label. No control arm, multiple cohorts.	Post ≥ 1 lines	Cohort 1: nivolumab (*n* = 74)	70.0	**39%**	44.2	13.8	-	~21% *	27% (9% D/C)	Phase II data of Nivolumab +/− Ipilimumab demonstrating high ORR, OS, PFS and DOR in MSI-H/dMMR mCRC. Indirect evidence that dual ICI may be superior at the expense of increased side effects.
			Cohort 2: nivolumab + ipilimumab (*n* = 119)	50.9	**65% (CR 13%)**	NR; 48 mo OS 71%	NR; 48 mo PFS 54%	NR	12%	32% (13% D/C)	
		1st line	Cohort 3: nivolumab + ipilimumab (*n* = 45)	52.4	**71%**	NR; 48 mo OS 72%	NR; 48 mo PFS 41%	NR	~13%	20% (16% D/C)	
		Post ≥ 1 lines	Nivolumab + relatlimab (*n* = 50)	47.4	**50%**	NR; 3 yr OS 56%	27.5	42.7	26%	14%	
Keynote 164 (14 September 2015) [96,101]	Phase II, multicenter, open-label. No control arm.	post ≥ 2 lines	Cohort A: Pembrolizumab (*n* = 61)	62.2	**32.8%** (4.9% CR)	31.4	2.3	NR	46%	16% (3% D/C)	Phase II data of Pembrolizumab demonstrated significant ORR and DOR in previously treated patients with MSI-H/dMMR mCRC.
		post ≥ 1 lines	Cohort B: Pembrolizumab (*n* = 63)	54.4	**34.9%** (14.3% CR)	47.0	4.1	NR	40%	13% (3% D/C)	
Keynote 177 (11 February 2016) [98,99,100]	Phase III, multicenter, open-label RCT (1:1 randomization). Crossover permitted.	1st line	Pembrolizumab (*n* = 153)	73.3	45.8% (17.6% CR)	**77.5**	**16.5**	75.4	30.1%	21.6%	Phase III data demonstrating Pembrolizumab improved OS, PFS, ORR, safety profile, and DOR in comparison to chemotherapy in MSI-H/dMMR mCRC.
		Comparator	5-FU based chemotherapy +/− targeted therapy (*n* = 154)	73.3	33.1% (4.5% CR)	**36.7**	**8.2**	10.6	12.3%	66.4%	
CheckMate-8HW ** (August 2019) [77,78]	Phase III, multicenter, RCT. 2:2:1 randomization to nivo/ipi, nivo alone, or ChT	1st line	Nivolumab + ipilimumab (*n* = 202)	31.5	-	-	**NR**	-	-	23%	First phase III trial demonstrates dual ICI with nivolumab and ipilimumab improves ORR, OS, PFS and DOR in comparison to both single-agent ICI and chemotherapy
		All lines of therapy	Nivolumab + ipilimumab (*n* = 296)	47	71% (30% CR)	-	**NR**	-	10%	22%	
			Chemotherapy (*n* = 101)	31.5	-	-	**5.9**	-	-	48%	
			Nivolumab alone (*n* = 286)	47	58% (28% CR)	-	**39.3**	-	19%	14%	
COMMIT (November 2017) [107]	Phase III open-label RCT, 1:1:1 randomization to FFX/bev/atezo, FFX/bev ***, or atezo alone	1st line	FOLFOX + bevacizumab + atezolizumab (*n* = 41)	3.5 yrs	80.6%	-	**30.0**	-	-	82.9%	Preliminary results suggest adding chemotherapy and bevacizumab to ICI improves efficacy outcomes
			Atezolizumab alone (*n* = 41)	3.5 yrs	46%	-	**4.3**	-	-	43.9%	

AE 3–4—incidence (%) of treatment-related grade 3–4 adverse events; ChT—chemotherapy; CR—complete response, DOR—duration of response (median in months unless otherwise specified), F/U—median follow-up, NR—not reached, PFS—progression free survival (median in months unless otherwise specified), PD—% of patients with progressive disease, ORR—objective response rate (%), OS—overall survival (median in months unless otherwise specified). The bolded outcome implies the primary outcome. * based on original publication. ** CheckMate-8HW results are for patients whose dMMR/MSI-H status was confirmed by central review and excluded those who were positive by local assessment only. Doses: for CheckMate-142, nivolumab monotherapy given 3 mg/kg q 2 weeks, nivolumab + ipilimumab given as nivolumab 3 mg/kg + ipilimumab 1 mg/kg q 3 weeks × 4 then nivolumab 3 mg/kg q 2 weeks; for Keynote trials, pembrolizumab given 200 mg q3 weeks × 35 doses; for CheckMate-8HW, nivolumab 240 mg + ipilimumab 1 mg/kg q 3 weeks × 4 doses then 480 mg q 4 weeks or nivolumab 240 mg q 2 weeks × 6 doses then 480 mg q 4 weeks. *** FFX/bev arm of COMMIT was closed due to results of Keynote-177; recruitment to other arms was suspended on 31 March 2025 after results of CheckMate-8HW were reported.

### 3.2. Dual Immunotherapy in Metastatic dMMR/MSI-H CRC

While the response rates in the dual immunotherapy CheckMate trials are numerically superior to those observed in Keynote-177, the major caveat is that primary analyses in the CheckMate trials were performed on patients with centrally confirmed dMMR/MSI-H disease, while Keynote-177 analyzed patients were based on local testing only and thus possibly included a larger number of patients with pMMR/MSS CRC [77,78,100,102]. Nevertheless, CheckMate-8HW did show an improved response rate in patients with centrally confirmed dMMR/MSI-H CRC to dual rather than single-agent immunotherapy (71% vs. 58%) and a lower incidence of primary progression (10% vs. 19%) although the rate of complete response was comparable (30% and 28%) [77,78]. Also of note, the response rates for all randomized patients are reported in the supplemental material for CheckMate-8HW and show an overall response rate of 63% to dual immunotherapy vs. 49% to nivolumab alone with rates of primary progression of 16% and 24%, respectively [77]. Taken together, these results imply that dual immunotherapy increases the response rate, but at this point it is unclear if survival or duration of response within responders is improved (data currently immature). It is conceivable that there is a population of patients who may not derive additional benefit from dual as opposed to single-agent immunotherapy; however, the subgroup analysis from this interim analysis was unable to clearly identify a group that did not seem to gain benefit from dual immunotherapy. More elderly patients and those with comorbidity are often selected for less intensive systemic therapies, and there is little data to help guide decision-making in these groups aside from patients ≥ 65 years seemingly deriving similar PFS benefits to younger patients in the dual immunotherapy arm of the CheckMate-8HW trial. Concerns have also been raised regarding the potential for increased toxicity with dual immunotherapy. In the CheckMate-8HW trial there were increased rates of grade 3/4 treatment-related serious adverse events with 16% in the dual arm compared with 7% in the single immunotherapy arm without differences in treatment-related deaths [77,78]. Consequently, there is increased toxicity in patients who receive dual as opposed to single-agent immunotherapy (see Table 2), but clinicians are limited in their ability to predict which patients are at greater risk of side effects, and the rates overall do not appear to be prohibitive. Part of the reduced toxicity when compared to trials of dual immunotherapy in other disease sites may be due to the lower dosing of ipilimumab (1 mg/kg vs. conventional 3 mg/kg) in the colorectal trials. Finally, the increased cost associated with dual immunotherapy will require important cost effectiveness evaluation once clinical trial data are mature. Given the improved response rates and early PFS results, dual immunotherapy has the potential to become a standard of care for dMMR mCRC. Future predictive biomarker studies are necessary in order to maximize the therapeutic threshold of immunotherapies in this setting.

### 3.3. Management of Hyperprogression and Mechanisms of Resistance

Hyperprogression is a term that has been used to describe rapid disease progression following the initiation of immunotherapy. In the Keynote-177 trial, the 30.1% incidence of primary disease progression was attributed to a combination of pseudoprogression, and misdiagnosis of pMMR/MSS disease and true biologic resistance to therapy [98,99,100].

Reasons for misdiagnosis and test failure have been explored in previous sections. It is notable that of the 14 patients in CheckMate-142 with hyperprogression, 6 did not appear to have dMMR/MSI-H disease on centralized review [98,110]. It is not clear if every patient who had dMMR/MSI-H disease by local analysis but pMMR/MSS disease on central review truly had pMMR/MSS disease, but it is likely that at least some portion did. Collectively, these results imply that at least some of the patients who experience hyperprogression on immunotherapy do so as the result of misdiagnosis.

Pseudoprogression is defined as an initial increase in radiographic disease burden followed by a subsequent observable response in the context of immunotherapy treatment. It may be due to immune-cell infiltration into the tumor causing an apparent increase in tumor size [111,112]. Unfortunately given that the most common way of diagnosing pseudoprogression is based on subsequent imaging, it is usually a retrospective diagnosis [111]. Indeed, in two retrospective studies including patients in both clinical trials and real-world, the incidence of pseudoprogression was ~10%. In these studies, 51.9% and 66.7% of patients with initial pseudoprogression went on to achieve a measurable response, and in one study, the 2-year PFS after initial pseudoprogression was 70.0% [113,114]. In the CheckMate-142 dual immunotherapy post-prior treatment cohort, authors note that 3 of the 14 patients with hyperprogression had an overall survival that exceeded four years [110]. Therefore, while clinicians must be vigilant for misdiagnosis, they should also be careful not to prematurely terminate a treatment that may be providing benefit based solely on first scans, especially in patients who are otherwise clinically stable or are showing other markers of improvement. Of note, pseudoprogression is most commonly seen within the first 3 months [113]. When available, additional markers such as circulating tumor DNA (ctDNA) and even repeat biopsy in high-stakes cases such as impending organ failure have been described as being helpful [111,115].

There are also documented cases of true biological resistance leading to initial treatment failure with immunotherapy. For example, in the Keynote 177 trial immunogenomics validation analysis by Bortolomeazzi et al., treatment response was associated with low Wnt activation, higher proportion of clonally expanded T cell populations, immunogenic mutations with high clonality, and inflammatory signaling deregulation via interferon-gamma pathways. In contrast, immune escape mechanisms leading to poor treatment response were mediated through alterations in antigen presentation and alterations in HLA class I expression [116].

More broadly, etiologies of primary resistance (i.e., no initial treatment response) can be classified into intrinsic and extrinsic causes. Intrinsic mechanisms are variable and reviewed in greater detail elsewhere but may include alterations in antigen presentation machinery, defects in immune sensing pathways such as JAK-STAT or cGAS-STING, immune suppression via upregulation of WNT signaling, RAS mutations, and heterogeneity in MMR protein loss [117,118,119]. Extrinsic factors may include VEGF-A overexpression, low MHC II expression, T-cell exhaustion driven by immunosuppressive chemokine-altering niche, and/or enhanced TGF-B signaling [120,121].

Acquired resistance, in contrast, is defined as disease progression after an initial period of response and is seen in up to 60% of patients. This is thought to be mediated by some combination of mechanisms leading to immune escape, such as mutations resulting in neoantigen depletion, acquired JAK1/2 mutations, antibiotic-mediated dysbiosis, selective pressure leading to resistant clones, upregulation of compensatory signals such as LAG-3, and T-cell exhaustion, amongst others. The full breadth and depth of discussion is beyond the scope of our present review, but these topics are reviewed in detail by Lei et al. [120] and Tufail et al. [121], respectively.

Ongoing research into predictive biomarkers may help better define populations that are at higher risk of resistance and/or those who may have better treatment response. Although not formally recommended as per NCCN, recent work has identified several emerging putative biomarkers such as TMB, proofreading domains of DNA polymerase ε and δ (POLE, POLD) mutations, and tumor microenvironment (TME) [122]. TMB correlates with response to ICI due to increased neoantigen production and consequently improved tumoral immunogenicity. POLE/POLD reduce proofreading during DNA replication and accelerate accumulation of somatic mutations leading to an “ultra-hypermutated” phenotype, which increases ICI-sensitivity through increased immune cell infiltration. Lastly, TME is known to modulate ICI responsiveness through effects on tumor-infiltrating lymphocytes (TILs), CD8+ T-cells, and inflammatory signaling. The mechanistic details of this are reviewed in Cataldi et al. [122].

Overall, treatment resistance poses a significant challenge in MSI-H/dMMR mCRC, and several strategies to circumvent these mechanisms are being explored including fecal microbiota transplant, escalation from single-agent to dual immunotherapy, and combinations with chemotherapies or targeted agents although none are standard of care at present [123,124]. Early data from the phase III randomized COMMIT study demonstrate the superiority of the combination of atezolizumab + FOLFOX + bevacizumab over atezolizumab alone with a response rate of 80.6% vs. 46%, suggesting that the addition of chemotherapy may circumvent some pathways of resistance, but recruitment to this trial has been suspended in light of the CheckMate-8HW results [107]. The phase II SEAMARK study is currently exploring concurrent targeting of BRAF V600E mutations with encorafenib and cetuximab along with pembrolizumab in patients with metastatic dMMR CRC harboring this mutation [125].

Collectively, these results suggest that when patients with dMMR CRC experience primary progression on immunotherapy, the clinician should have a low threshold to consider the misdiagnosis of pMMR disease. Furthermore, they should consider the entirety of the clinical picture and have a high degree of suspicion for pseudoprogression. If there appears to be true disease resistance, then there are a variety of experimental approaches being explored, and enrollment in clinical trials should be considered.

### 3.4. Progression on Immunotherapy: Role for Escalation, Rechallenge, and Retreatment

Given the potential of dual immunotherapy to produce higher response rates compared to single-agent immunotherapy, a key question is whether there is a role for escalation, either at progression or empirically, in those who were previously treated with single-agent immunotherapy. Large-scale randomized control data in this domain is limited. In Keynote-177, 13.1% of patients who progressed on single-agent immunotherapy received further immunotherapy as a component of their subsequent therapy, although this does not appear to have been dual immunotherapy and outcomes of this patient group are not specifically reported [98,99]. Immunotherapy was not one of the prior lines of therapy in patients receiving dual immunotherapy in the previously treated cohort of CheckMate-142 and patients in the nivolumab arm did not crossover to ipilimumab + nivolumab in CheckMate-8HW [77,110].

In 2022, Kasi et al. published a case series of 3 patients who were rechallenged with ipilimumab + nivolumab after progression on pembrolizumab and experienced clinical response and reduction in circulating tumor DNA [126]. Similarly, Hamre et al. described a case of a patient with synchronous renal cell carcinoma and metastatic dMMR CRC whose disease stabilized on retreatment with ipilimumab + nivolumab after progressing on pembrolizumab [127]. Chen et al. performed a retrospective study on ideal treatment post-immunotherapy in 51 patients with MSI-H gastrointestinal cancers, including 35 patients with CRC. Patients were treated with either chemotherapy + targeted therapy (*n* = 25) or anti-PD-1/PD-L1 therapy along with another agent (*n* = 26; including a CTLA-4 inhibitor in 2 patients). They showed that the patients who received immunotherapy as part of their second-line treatment did better (disease control rate 80.8% vs. 44.0%, median PFS 6.9 mo vs. 3.0 mo, and median overall survival NR vs. 14.1 mo) than those who received chemotherapy as the backbone [128]. Collectively, these results suggest that in patients with metastatic dMMR CRC who experience progression after treatment with single-agent immunotherapy, there may be benefit to escalation of treatment with a further immunotherapy containing regimen. Simmons et al. showed in 2023 that 56 out of 64 patients with advanced CRC who received a median of 17.6 months of immunotherapy had ongoing disease control after stopping treatment. In the 8 patients who developed progression, 7 were successfully rechallenged with an immunotherapy regimen, while the eighth could not be retreated due to toxicity [129]. This study supports a role for re-exposure to immunotherapy in patients who progress while off treatment. While there is little data to support escalation to dual immunotherapy in patients who are responding to single-agent immunotherapy, the above series provides some support for escalation of immunotherapy at time of progression or re-challenging in the case of a prolonged treatment-free interval, but the field would benefit from reports with larger sample sizes.

Molecular targeting of abnormalities such as BRAF V600E or NTRK fusions should be considered at time of progression. Although randomized trials have not investigated this question specifically, some patients in targeted therapy trials have also harbored dMMR/MSI-H disease. For example, 41 of 444 patients with BRAF V600E metastatic CRC who received a regimen containing encorafenib + cetuximab in the second line in the BEACON trial also harbored MSI-H disease and appeared to respond favorably [130]. Cases of good responses to targeted therapies following progression on immunotherapy for dMMR/MSI-H CRC have also been reported, and there is a strong biological rationale for this approach while we await larger studies [131,132]. Whether an approach employing immunotherapy with targeted therapy in the first line results in improved overall survival compared to reserving targeted therapy for time of progression also remains an unanswered question.

## 4. Management of Localized dMMR/MSI-H Colorectal Cancer

In the localized setting, dMMR or MSI-H status has been recognized as a predictor of poor response and even potential harm from treatment with neoadjuvant and adjuvant fluorouracil-based chemotherapies, although better prognosis overall [11,133,134]. Indeed, some guidelines suggest that dMMR/MSI-H status should be used to recommend against adjuvant chemotherapy in early-stage colon cancers [135]. Given the poor response of these tumors to adjuvant chemotherapy but the positive results seen with immunotherapy in metastatic dMMR/MSI-H CRC, ongoing trials have sought to investigate the role for neo/adjuvant immunotherapy in localized dMMR/MSI-H CRC. Key trials of adjuvant and neoadjuvant immunotherapy in this setting are described in Table 3. Recently, results of the randomized controlled phase III ATOMIC RCT were published. The trial randomized patients with surgically resected stage III dMMR/MSI-H colon cancers to an experimental group consisting of 12 cycles of biweekly FOLFOX chemotherapy with atezolizumab plus an additional 13 cycles of atezolizumab while the control group received 12 cycles of FOLFOX chemotherapy only as adjuvant therapy. At 40.9 months, patients in the chemo-immuno experimental arm had better 3 year disease-free survival compared to those in the chemotherapy alone control arm (86.3% vs. 76.2%) [136,137]. An ongoing phase II study is investigating the role for adjuvant toripalimab monotherapy in patients with resected dMMR/MSI-H colon cancers that are stage IIB, IIC, and III [138].

While the available results of adjuvant trials are promising, there has also been significant interest in a neoadjuvant approach, particularly in rectal cancer, where surgeries can be highly morbid with a significant impact on quality of life. In the pivotal work by Cercek et al., all 49 patients with dMMR rectal cancer who were treated with 6 months of dostarlimab had a clinical complete response (cCR) and proceeded to non-operative management. This response was sustained in 37 patients who avoided surgery altogether. Importantly, there appeared to be concordance of the defined response evaluations between endoscopic and MRI findings. In the entire group, the recurrence free-survival was 96% [139]. The phase II AZUR 1 trial (NCT05723562) of dostarlimab in rectal cancer recently completed accrual of 154 patients, and results from this study are eagerly awaited since small studies are prone to selection bias and longer-term follow-up is necessary to evaluate the potential of delayed recurrence [140]. An area of clinical uncertainty pertains to radiographic response to immunotherapy for dMMR colorectal cancers. For example, a radiographic complete response rate of 33.3% was seen in the small (*n* = 6) cohort of patients with rectal cancer who pursued a non-operative approach after 6 months of pembrolizumab in a study by Ludford et al. [141,142]. However, several patients in this study with dMMR colon cancer who did not achieve a clinical response were found to have a pathological complete response (i.e., absence of viable tumor tissue) on pathology, highlighting that the prediction of true biological response based on clinically assessable parameters is imperfect [141]. As a result, surgical decision-making is challenging in the setting of neoadjuvant immunotherapy as evidenced by the discrepancies in endoscopic and imaging techniques. In a retrospective analysis of dMMR/MSI-H CRC patients that received PD-1 therapies, there was a discrepancy in response assessment in 54% of the cases between endoscopy and imaging and many patients were found to have irregularities on these modalities despite eventually having confirmed pCR at the time of surgery [143]. Clearly, a refinement of the response assessment criteria is required prior to employing non-operative management (NOM) strategies in colon cancer, while rectal cancers response appears to be more consistent, at least in the clinical trial setting. Further concerns with a neoadjuvant/non-operative approach revolve around the durability of responses. In the setting of rectal cancer, much of the survival data is immature (see Table 3) and from small trials, but overall results appear promising; however, long term follow-up from these trials (and evaluation of the NOM surveillance strategy) will be needed to reassure the oncology community of the lasting benefits of this approach [139,144,145]. Presently, there is also no randomized phase III evidence to inform this treatment strategy, although the AZUR-2 trial of neoadjuvant dostarlimab in resectable dMMR colon cancers that are stage III or cT4 is actively recruiting (NCT05855200). In this trial, patients are randomized 2:1 to peri-operative dostarlimab vs. standard of care adjuvant chemotherapy, with the primary endpoint being event-free survival and pathological response; overall survival and safety are key secondary endpoints [146,147]. This trial is incorporating circulating tumor DNA assessments, which may prove valuable in predicting response to neoadjuvant immunotherapies since current endoscopic and imaging techniques may be discordant [147].

Several neoadjuvant immunotherapy trials have specifically investigated or included patients with dMMR colon cancers, and results in this domain also appear encouraging. Pathologic complete response rates of 44% and 68% have been observed in the largest of these trials (for rates in other trials, see Table 3) [148,149,150]. There is emerging evidence that there is a direct link between the duration of neoadjuvant therapy and chances of complete response [151]. Indeed, this appears to be consistent with the studies listed in Table 3, where the best results were obtained with either 6 months of neoadjuvant immunotherapy or the use of dual immunotherapy. Although survival data are immature, the substantial improvement in disease-free survival seen in the NICHE-2 study compared with what would generally be expected for dMMR/MSI-H colon cancers treated with conventional neo/adjuvant chemotherapy has led some investigators to propose that randomized trials in this context would be unethical due to lack of clinical equipoise [150].

Taken together, these data suggest that immunotherapy (whether neoadjuvant or adjuvant) is an important emerging strategy for the treatment of localized dMMR/MSI-H colorectal cancers. Whether this takes the form of adjuvant or neoadjuvant treatment remains to be seen, although there is biological rationale for the latter [152]. Furthermore, neoadjuvant treatment offers the possibility of reducing morbidity from surgery or, in some cases, avoiding surgery altogether. Long-term survival data from patients managed with a non-operative approach will be important in determining the feasibility of this mode of treatment for localized rectal as well as colon cancers. These trials have generally demonstrated a favorable safety profile (see Table 3) with only infrequent delays in surgery, suggesting that this strategy is promising and further clinical trial refinements may allow for more non-operative approaches in the future.

**Table 3:** Key Trials of Immunotherapy in Patients with Localized dMMR/MSI-H Colon or Rectal Cancer. References [136,137,139,140,141,142,144,145,146,148,149,150,153,154,155,156,157,158,159,160,161,162,163,164].

Trial (Enrollment Start Date)	Study Design	Group	Disease Site	Neoadjuvant Treatment	Surgery?	Adjuvant Treatment	Follow up (Months)	CR Rate (P/R)	DFS (D)/RFS (R)	OS	AE (Gr 3+)
Voltage (January 2017) [144,154]	Phase II, multi-cohort	dMMR Cohort (*n* = 5)	Rectal	Capecitabine + radiation + 5 cycles nivolumab	Yes	+/−adjuvant FOLFOX (1 patient)	56.4	P: 60%	R: 100% at 3 yrs	100% at 3 yrs	0%
NICHE-2 (March 2017) [150,155]	Phase II, single arm	Study cohort (*n* = 111)	Colon	Nivolumab × 2 doses, ipilimumab × 1 dose	Yes	+/−adjuvant chemotherapy	26	P: 68%	D: 100% at 26.2 mo	-	4%
ATOMIC (September 2017) [136,137]	Phase III RCT	Experimental (*n* = 355)	Colon	-	Yes	Atezolizumab + FOLFOX	40.9	-	D: 86.3% at 3 yrs	-	84.1%
		Control (*n* = 357)		-		FOLFOX		-	D: 76.2% at 3 yrs	-	71.9%
NICOLE (June 2018) [153]	Phase II, multi-cohort	dMMR (*n* = 3)	Colon	Nivolumab × 2 doses	Yes	-	N/R	P: 0%	-	-	N/R
Ludford (October 2019) * [141,142]	Phase II, multi-cohort	Colon, operative (*n* = 12)	Colon	Pembrolizumab × 6 mo	Yes	-	U/S *	P: 83.3%	N/R *	N/R *	9% in entire study cohort *
		Colon, non-op (*n* = 7)	Colon		No	+/−Continue pembro to 1 yr		R: 42.9%			
		Rectal, operative (*n* = 2)	Rectal		Yes	-		P: 50%			
		Rectal, non-op (*n* = 6)	Rectal		No	+/−Continue pembro to 1 yr		R: 33.3%			
Chen (October 2019) [163]	Phase II	Non-operative	Rectal	Sintilimab × 4 cycles +/− 4 more cycles	No (if PCR)	-	17.2	**	D: 100% at 17.2 mo	100% at 17.2 mo	6%
		Operative (*n* = 6)			Yes	+/−4 cycles sintilimab +/− CapeOX		P: 50%			
Cercek (December 2019) [139,156]	Phase II, multi-cohort	Rectal (*n* = 49)	Rectal	Dostarlimab × 6 mo	No	-	30.2	R: 100%	R: 96%	-	2.0%
		Colon (*n* = 22)	Colon		If incomplete response	-	U/S	R: 82%	R: 100%	-	9.1%
PICC (May 2019) [145,157]	Phase II, multi-cohort	Cohort 1 (*n* = 24)	Colon/Rectum	Toripalimab × 6 cycles	Yes	Continuation of neoadjuvant treatment × 6 months	14.9	P: 75%	D: 85%	91%	1.5%
		Cohort 2 (*n* = 23)		Toripalimab × 6 cycles + celecoxib				P: 87%	D: 100%	100%	1.5%
NEOCAP (September 2020) [160]	Phase II	Colon (*n* = 40)	Colon	4 cycles camrelizumab + apatinib ***	Yes, but if CR had the option for non-operative	At investigator’s discretion (includes continue anti-PD-1 +/− apatinib vs. observation)	16.4	73% (38/52) P: 61% (13/23) ***	-	-	38% (including 1 death)
		Multiple primary (*n* = 6)	Rectum								
		Rectum (*n* = 6)	Multiple								
IMHOTEP (December 2021) [161,162]	Phase II	Colon (*n* = 72)	Colon	Pembrolizumab; 1 or 2 cycles	Yes; 11 did not receive surgery	Pembrolizumab × 1 year	U/S	P: 55.4% (36/65)	-	-	9.2%
		Rectum (*n* = 15)	Rectum					P: 45.5% (5/11)	-	-	
NEOPRISM-CRC (July 2022) [158]	Phase II, single arm	Study group (*n* = 32)	Colon	Pembrolizumab × 3 cycles	Yes	-	6	P: 53%	D: 100%	-	0%
NICHE-3 (December 2022) [149]	Phase II, single arm	Study group (*n* = 59)	Colon	2 cycles of nivolumab + relatimab	Yes	+/−adjuvant chemotherapy	8	P: 68%	D: 98% at 8 mo	100% at 8 mo	10%
RESECT-C (February 2023) [148,159]	Phase II, single arm	Study group (*n* = 85)	Colon	Pembrolizumab × 1 cycle	Yes (1 did not)	-	U/S	P: 44%	N/R	N/R	8% ^t^
AZUR-1 (April 2023) [140]	Phase II, single arm	Study group	Rectal	Dostarlimab × 9 cycles	Y/N (depends on CR)	-	-	-	-	-	-
Wang et al. (May 2023) [164]	Phase Ib, randomized	Single IO (*n* = 49)	Colon	Sintillimab × 2 cycles	Y (45/49)		21.4	P: 46.7%	D: 100% at 21.4 mo	98% at 21.4 mo	18.4%
		Dual IO (*n* = 52)		IBID10 × 1 cycle + Sintillimab × 2 cycles	Y (51/52)			P: 78.4%	D: 100% at 21.4 mo	98% at 21.4 mo	30.8%
AZUR-2 (August 2023) [146]	Phase III, RCT	Immunotherapy	Colon	Dostarlimab	Yes	Dostarlimab	-	-	-	-	-
		Standard		-		FOLFOX/CAPEOX vs. watch and wait					

AE (Gr3+)—% of patients experience adverse event grade 3 or higher CR rate (P/R)—complete response rate where P indicates pathologic and R indicates radiographic, DFS/RFS—disease-free survival or relapse-free survival where D indicates DFS and R indicated RFS, OS—overall survival; U/S—unspecified. * In the Ludford study, median follow-up for patients in operative/non-operative groups with colon and rectal cancer was not specified for these subgroups; DFS and OS were not specified for colon and rectal cancers individually, but in the entire group (including patients with pancreas, gastric, endometrial, duodenal, brain, and ampullary cancers) who were managed operatively, the 3-yr EFS rate was 83% and 3-yr OS was 100%, while 3-yr EFS was 76% and 3-yr OS was 89% in patients who did not undergo surgery. ** clinical response was 75% (12/16) in all patients; in the patients who received surgery (6), pCR rate was 50% (3/6). *** in patients with colon cancer, treatment could be extended by four more cycles if a clinical response was observed to initial treatment in order to attempt to obtain a pCR and proceed with nonoperative management; of 52 patients, 28 patients had CR, of which 24 went on to watch and wait. In the 24 patients not having CR, 19 had surgery with no complete response. ^t^ Two deaths were reported post-operatively in addition to 7/85 patients experiencing Gr 3 AEs.

## 5. Conclusions

Immunotherapy has drastically changed the management of dMMR/MSI-H CRC and all patients with a diagnosis of CRC should be assessed for dMMR/MSI-H status. The current standard is assessment via IHC for dMMR with PCR for MSI-H status as an acceptable alternative or complementary test. Treating clinicians should be aware of the MMR patterns that suggest Lynch syndrome, and these patients, along with those who have a strong family history or other risk factors should be referred for germline testing. Identification of BRAF and/or MLH1 loss should prompt testing of methylation status. Finally, clinicians should be aware of the limitations of dMMR/MSI-H testing.

In the metastatic setting, dual immunotherapy may become a standard of care. Whether its advantage over single-agent immunotherapy is primarily in increasing response rates or whether responses will also be deepened by this strategy remains to be seen, and mature survival data from dual immunotherapy trials are awaited. At the time of disease progression, there may be a role for rechallenging with immunotherapy depending on the clinical context, and physicians should be aware of the targetable mutations associated with dMMR/MSI-H CRC that may inform second-line treatments.

For localized dMMR/MSI-H CRC, there is mounting evidence for the role of immunotherapy in either the adjuvant or neoadjuvant setting. Neoadjuvant approaches are gaining popularity, and early evidence suggests that the duration of immunotherapy is an important determinant of clinical response. In the future fully non-operative strategies may become feasible provided that appropriate mechanisms are in place to accurately detect disease response, growth, or regrowth and not miss a “window of opportunity” for surgery. Results from ongoing studies exploring neoadjuvant and non-operative management are eagerly awaited.

## Figures and Tables

**Table 1:** Comparison of Diagnostic Methods for Detecting dMMR/MSI in CRC.

Test	Advantages	Limitations	Sensitivity/Specificity	Typical Clinical Application
Immunohistochemistry (IHC) for MMR proteins	-rapid/most available test -highly accurate if testing is conducted for all four proteins - inexpensive -may provide clue to sporadic vs. inherited	-may detect nonfunctional proteins -protein expression can change following treatment (i.e., MSH6 in rectal cancer following radiation)	Sensitivity: 85–100% [61] Specificity: 85–92% [61]	-most common first line test
Polymerase chain reaction (PCR) for MSI	-current gold standard for predicting immunotherapy responsiveness	-requires healthy tissue -more costly -longer turnaround time than IHC/technical expertise -does not provide information on affected protein/may require follow-up testing	Sensitivity: 95.6% [62,63] Specificity: 100% [62,63]	-alternative or complementary test to IHC
Next generation sequencing	-concurrent identification of mutations of interest, quantification of tumor mutational burden	-increased cost relative to PCR and IHC -limited availability -longer turnaround time and requires more tissue	Variable depending on approach; see reference [63]	-Use currently limited by accessibility

Summarized from references [54,55,61,62,63].

## Data Availability

No new data were created or analyzed in this study. Data sharing is not applicable to this article.
